# Treatment With Gemfibrozil Prevents the Progression of Chronic Kidney Disease in Obese Dahl Salt-Sensitive Rats

**DOI:** 10.3389/fphys.2020.566403

**Published:** 2020-09-18

**Authors:** Corbin A. Shields, Bibek Poudel, Kasi C. McPherson, Andrea K. Brown, Ubong S. Ekperikpe, Evan Browning, Lamari Sutton, Denise C. Cornelius, Jan M. Williams

**Affiliations:** ^1^Department of Experimental Therapeutics and Pharmacology, University of Mississippi Medical Center, Jackson, MS, United States; ^2^Department of Emergency Medicine, University of Mississippi Medical Center, Jackson, MS, United States

**Keywords:** obesity, renal injury, SS rat, SS^LepR^mutant rat, lipid accumulation, gemfibrozil

## Abstract

Recently, we reported that Dahl salt-sensitive leptin receptor mutant (SS^LepR^mutant) rats exhibit dyslipidemia and renal lipid accumulation independent of hyperglycemia that progresses to chronic kidney disease (CKD). Therefore, in the current study, we examined the effects of gemfibrozil, a lipid-lowering drug (200 mg/kg/day, orally), on the progression of renal injury in SS and SS^LepR^mutant rats for 4 weeks starting at 12 weeks of age. Plasma triglyceride levels were markedly elevated in the SS^LepR^mutant strain compared to SS rats (1193 ± 243 and 98 ± 16 mg/day, respectively). Gemfibrozil treatment only reduced plasma triglycerides in the SS^LepR^mutant strain (410 ± 79 mg/dL). MAP was significantly higher in the SS^LepR^mutant strain vs. SS rats at the end of the study (198 ± 7 vs. 165 ± 7 mmHg, respectively). Administration of gemfibrozil only lowered MAP in SS^LepR^mutant rats (163 ± 8 mmHg). During the course of the study, proteinuria increased to 125 ± 22 mg/day in SS rats. However, proteinuria did not change in the SS^LepR^mutant strain and remained near baseline (693 ± 58 mg/day). Interestingly, treatment with gemfibrozil increased the progression of proteinuria by 77% in the SS^LepR^mutant strain without affecting proteinuria in SS rats. The renal injury in the SS^LepR^mutant strain progressed to CKD. Moreover, the kidneys from SS^LepR^mutant rats displayed significant glomerular injury with mesangial expansion and increased renal lipid accumulation and fibrosis compared to SS rats. Treatment with gemfibrozil significantly reduced glomerular injury and lipid accumulation and improved renal function. These data indicate that reducing plasma triglyceride levels with gemfibrozil inhibits hypertension and CKD associated with obesity in SS^LepR^mutant rats.

## Introduction

The incidence of obesity has increased considerably within the last decade and is now considered an independent risk factor for chronic kidney disease (CKD) (Chagnac et al., 2000; Bosma et al., 2004; Ejerblad et al., 2006; Hsu et al., 2006; Jacobs et al., 2010). One of the hallmark characteristics of obesity is dyslipidemia (National Cholesterol Education Program Expert Panel on Detection Evaluation, and Treatment of High Blood Cholesterol in Aults, 2002; Huang, 2009). Moreover, the presence of dyslipidemia has been associated with a greater risk for the development of CKD (Kasiske et al., 1993; Guijarro and Keane, 1996; Keane, 2000). In support of this finding, preclinical studies have demonstrated similar results, in which glomerular injury and tubulointerstitial damage were markedly increased in the setting of dyslipidemia (McPherson et al., 2016, 2020). We have previously reported that the obese Dahl salt-sensitive leptin receptor mutant (SS^LepR^mutant) rat develops dyslipidemia, progressive proteinuria, and glomerular injury as early as 6 weeks of age independent of hyperglycemia and elevations in arterial pressure (McPherson et al., 2016, 2020). Moreover, the kidneys from SS^LepR^mutant rats displayed lipid accumulation, which is one of the most common characteristics of renal disease associated with obesity at this same time period (McPherson et al., 2020). Previous studies have provided evidence that renal lipid accumulation leads to structural and functional changes in glomeruli and tubules that lead to proteinuria and renal dysfunction (Foster et al., 2011; Straub et al., 2013; de Vries et al., 2014; DeZwaan-McCabe et al., 2017; Praga and Morales, 2017; Jonker et al., 2018). Additionally, lipid accumulation in the kidney contributes to oxidative stress, inflammation, and fibrosis (Yang et al., 2017), in which all of these processes contribute to the development of CKD. Recently, we observed that plasma triglyceride levels were substantially higher in the SS^LepR^mutant strain compared to their lean wild-type counterparts (McPherson et al., 2016, 2020), which was linked to alterations in various lipid transporters that may have led to significant renal lipid accumulation in the SS^LepR^mutant strain during the development of CKD (McPherson et al., 2020). However, it remains to be determined whether the increased triglyceride levels contribute to the progression of renal disease in the SS^LepR^mutant strain.

Lipid-lowering drugs are commonly administered to patients suffering from various forms of CKD with dyslipidemia (Kasiske, 1998). However, the link between dyslipidemia and the progression of renal disease has been inconsistent and determining the role of dyslipidemia as a mediator of CKD remains unclear (Samuelsson et al., 1997; Hadjadj et al., 2004; Dalrymple and Kaysen, 2008; Rahman et al., 2008; Kaysen, 2009; Navaneethan et al., 2009; Chawla et al., 2010). While the statins are more effective in reducing plasma cholesterol concentration, the fibrates are more successful in decreasing the levels of triglycerides. However, one of the major disadvantages of fibrates is that this class of drugs can lead to a reduction in renal function by potentially inhibiting the synthesis of vasodilatory prostaglandins (Wilson et al., 1995; Ledwith et al., 1997). One of the fibrates in particular, gemfibrozil, appears to be free of this renal dysfunction effect (Broeders et al., 2000). Therefore, the objective of the current study was to examine the effects of gemfibrozil treatment on the progression of renal injury and CKD in SS^LepR^mutant rats. We hypothesized that treatment with gemfibrozil would decrease plasma triglyceride levels, reduce renal lipid accumulation, and prevent the progression of CKD in the obese SS^LepR^mutant strain.

## Materials and Methods

### General

Experiments were performed on a total of 54 female and male SS and SS^LepR^mutant rats at 12 weeks of age. SS and SS^LepR^mutant strains were obtained from our in-house colony of heterozygous SS^LepR^mutant rats, which were created using zinc-finger nuclease technology as previously described (McPherson et al., 2016). Genotyping was performed by the Molecular and Genomics Facility at the University of Mississippi Medical Center. The SS^LepR^mutant rat develops renal injury associated with obesity without hyperglycemia (McPherson et al., 2016, 2020). We pooled a small number of female SS and SS^LepR^mutant rats with the male rats in the current study, since the female rats display a similar susceptibility to renal injury as their male counterparts from our colony, which is highlighted in Table 1. Food and water were provided *ad libitum* throughout the study. Rats were maintained on a 1% NaCl diet (TD8640; Harlan Laboratories, Madison, WI, United States) at wean. The rats were housed in the Laboratory Animal Facility at the University of Mississippi Medical Center was approved by the American Association for the Accreditation of Laboratory Animal Care, and all protocols were approved by the University of Mississippi Medical Center Institutional Animal Care and Use Committee.

**TABLE 1:** Comparison of metabolic and cardiovascular parameters in female and male Dahl salt-sensitive (SS) and SS leptin receptor mutant (SS^LepR^mutant) rats between 12 and 14 weeks of age.

Metabolic parameters	SS		SS^LepR^mutant	
		
	Female	Male	Female	Male
Body weight (g)	210 ± 3	347 ± 5^#^	392 ± 4^†^	471 ± 15^†^^#^
Glucose (mg/dL)	96 ± 4	102 ± 6	104 ± 4	100 ± 4
MAP (mmHg)	135 ± 5	133 ± 6	132 ± 17	147 ± 13
Proteinuria (mg/day)	41 ± 6	94 ± 19	500 ± 60^†^	552 ± 53^†^

*Values are means ± SE. ^†^ P <0.05 vs. SS rats within the same sex and ^#^P <0.05 vs. female rats within the same strain (n = 6–17 per group in each parameter).*

***Protocol 1: Comparison of body weight, blood glucose, arterial pressure, and proteinuria in female and male SS and SS^LepR^mutant rats***. Experiments were performed on 12–14 week-old female and male SS and SS^LepR^mutant rats. The rats were weighed and placed in metabolic cages for an overnight urine collection to determine proteinuria using the Bradford method (Bio-Rad Laboratories; Hercules, CA, United States), and a blood sample was collected from the tail vein for the measurement of blood glucose levels (glucometer from Bayer HealthCare; Mishawaka, IN, United States). Mean arterial pressure (MAP) was measured in conscious animals via the tail-cuff method (MC4000 BP Analysis System, Hatteras Instruments, Cary, NC, United States). One week prior to measuring MAP the rats were trained and adapted to restraint for 15–25 min for 3 sequential days, and MAP was measured at the same time of day. These experiments were performed to justify that female SS^LepR^mutant rats had a comparable susceptibility to renal injury as the male SS^LepR^mutant rats.

***Protocol 2: Effects of Gemfibrozil on the progression of renal injury in SS and SS^LepR^mutant rats***. Experiments were performed on 12 week-old SS and SS^LepR^mutant rats. The measurement of proteinuria and blood glucose levels as described in Protocol 1. After collecting baseline data, SS and SS^LepR^mutant rats were separated into four groups: (1) SS and (2) SS^LepR^mutant rats were treated with vehicle – powdered food (TD8640) and (3) SS and (4) SS^LepR^mutant rats were treated with gemfibrozil (200 mg/kg/day, orally in the powdered food) for 4 weeks. Every 2 weeks rats were placed in metabolic cages until the rats reached 16 weeks of age, and proteinuria and blood glucose levels were measured at each time period. During the final week of the study, rats were placed under anesthesia, and a catheter was inserted in the carotid artery for the measurement of MAP. After a 24 h recovery period, catheters were connected to pressure transducers (MLT0699; ADInstruments, Colorado Springs, CO, United States) coupled to a computerized data PowerLab acquisition system (ADInstruments, Colorado Springs, CO, United States). MAP was recorded continuously for 30 min after a 30 min equilibration period. After arterial pressure measurements, a final blood sample was taken from the abdominal aorta to measure plasma triglyceride and total cholesterol concentrations (Cayman Chemical Company, Ann Arbor, MI, United States) and insulin (Mercodia Rat Insulin ELISA, Uppsala, Sweden). Next, both kidneys were weighed and each kidney was placed in individual flasks containing a 10% buffered formalin fixation solution for histology.

### Renal Histopathology and Lipid Accumulation

#### Measurement of Glomerular Injury and Renal Fibrosis

Paraffin kidney sections were prepared from the right kidneys collected from SS and SS^LepR^mutant rats treated with and without gemfibrozil. Kidney sections were cut into 3 μm sections and stained with Periodic acid-Schiff (PAS) and Masson’s Trichrome. To determine glomerular injury, 30 glomeruli per PAS section were scored in a blinded fashion on a 0–4 scale with 0 representing a normal glomerulus, 1 representing a 25% of loss, 2 representing a 50% loss, 3 representing a 75% loss, and 4 representing >75% loss of capillaries in the tuft. To determine the degree of renal fibrosis, 5–10 representative images per section was captured using a SeBa microscope equipped with a color camera (Laxco Inc., North Creek, Washington, United States) and analyzed for the percentage of the image stained blue (primarily collagen) in the Masson’s trichrome-stained sections using NIS-Elements D 3.0 software.

#### Measurement of Renal Lipid Accumulation and Low-Density Lipoprotein (LDL)

To determine renal lipid accumulation via oil-red-O staining, the left kidney was cut in half, removed from the 10% buffered formalin solution, and washed in 0.1% PBS for 5 h. The other half was placed back into the 10% buffered formalin solution for later use. The kidneys were then placed in 30% sucrose overnight or until the kidneys sunk to the bottom of the container. Kidneys were then cut into 10 μm sections and stored at −80°C overnight. On the next day, the frozen sections were thawed and allowed to air dry for 20 min before fixation in 40% formalin and washed with H_2_O. Then, the sections were stained in Oil-red-O solution (EKI; Joilet, IL, United States) for 10 min and washed with H_2_O. The sections then were counterstained with Harris hematoxylin containing acetic acid (Stat Lab; McKinney, TX, United States) for 1 min and washed again with H_2_O. Finally, the sections were incubated in ammonia water to cause the blue counterstain and washed a final time with H_2_O before mounting with an aqueous mounting medium (Thermo Fisher Scientific, Waltham, MA, United States). Images were obtained using the same microscope as mentioned above and analyzed for the percentage of the image stained red using the NIS-Elements D 3.0 software.

To measure renal LDL accumulation, paraffin-embedded sections (3 μm) were deparaffinized with xylene, followed by dehydration utilizing a series of ethanol with decreasing concentrations keeping the sections at 60°C for 30 min. Then, the sections were incubated in a citrate buffer (pH-6.0) solution for 20 min using a microwave oven to retrieve the LDL receptor in the kidney section. The sections were washed 3 times with DPBS containing Ca^2+^ and Mg^2+^ (Sigma-Aldrich, St. Louis, MO, United States) for 5 min and incubated in 10% blocking goat serum solution for 2 h at room temperature. After removal of the blocking solution, the sections were incubated with FITC-labeled BODIPY FL LDL (Life Technologies Corporation, Carlsbad, CA, United States) overnight at 4°C. Then, the sections were washed three times with DPBS containing Ca^2+^ and Mg^2+^ for 5 min and incubated with 0.001% Evans blue for 10 min to quench auto-fluorescence. Next, the sections were washed three times with DPBS containing Ca^2+^ and Mg^2+^ for 5 min. The slides were then applied with a drop of an anti-fade mounting medium with DAPI (H-1200, Vector Laboratories, Inc., Burlingame, CA, United States) and cover slipped. Images were obtained using a Nikon Eclipse 55i microscope equipped with a Nikon DS-Fi1 color camera (Nikon, Melville, NY, United States).

### Statistical Analysis

Statistical analysis was performed using GraphPad Prism 8 (GraphPad Software, San Diego, CA, United States). The significance of the difference in mean values for a single time point was determined by an one-way ANOVA followed by the Tukey’s multiple comparisons test. Temporal changes in metabolic and cardiovascular parameters were compared between and within strains using a two-way ANOVA followed by the Holm-Sidak test. A *p*-value <0.05 was considered significantly different. The data are presented as mean ± SEM.

## Results

### Sex Differences in Female and Male SS and SS^LepR^Mutant Rats

The comparison of metabolic and cardiovascular parameters in female and male SS and SS^LepR^mutant rats is presented in Table 1. When examining body weight, both male SS and SS^LepR^mutant rats had a significantly higher body weight when compared to their female counterparts (210 ± 3 vs. 347 ± 5 and 392 ± 4 vs. 471 ± 15 g, respectively). We did not observe any sex or strain differences in blood glucose levels in SS and SS^LepR^mutant rats. Similar to blood glucose, MAP was not different among the groups. Proteinuria had a tendency to be elevated in male SS rats compared to the values seen in female SS rats (94 ± 19 vs. 41 ± 6 mg/day), but it did not reach statistical significance. While we did not observe any significant differences in proteinuria between female and male SS^LepR^mutant rats (500 ± 60 vs. 552 ± 53 mg/day, respectively), proteinuria was markedly elevated in the SS^LepR^mutant strain compared to their SS littermates.

### Metabolic Parameters

**FIGURE 1:**
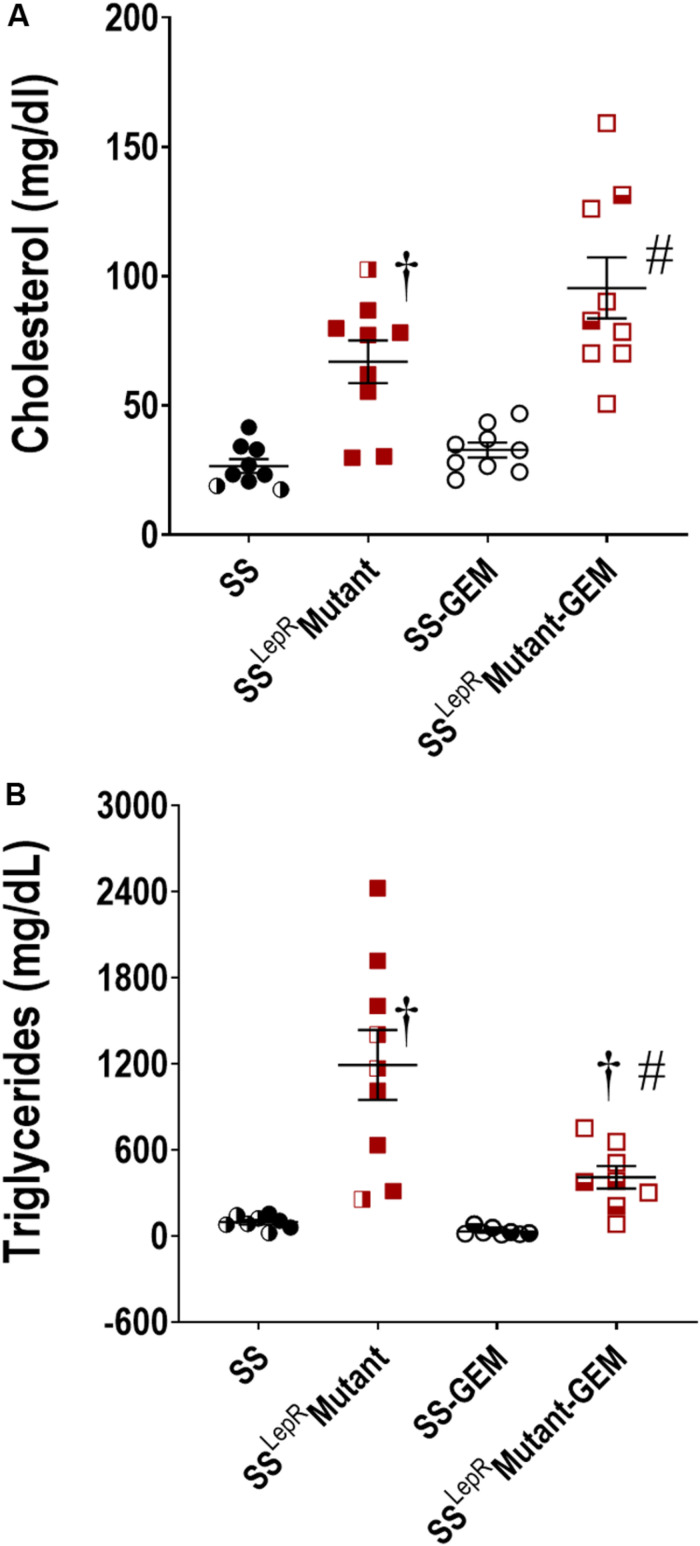
Efficacy of gemfibrozil treatment on plasma cholesterol **(A)** and triglyceride **(B)** levels in wild-type Dahl salt-sensitive (SS) rats and the obese SS leptin receptor mutant (SS^LepR^mutant) strain. Numbers of rats studied (*n* = 7–9 per group). Female rats in each group are represented by partially filled symbols. Values are means ± SE. ^†^*p* <0.05 vs. SS rats within the same treatment, and ^#^*p* <0.05 vs. vehicle-treated rats within the same strain.

The efficacy of gemfibrozil on plasma cholesterol and triglyceride levels in SS and SS^LepR^mutant rats is presented in Figure 1. In Figure 1A, cholesterol was significantly elevated in SS^LepR^mutant rats compared to SS rats (67 ± 8 and 27 ± 3 mg/day, respectively), and chronic treatment with gemfibrozil did not have an effect on cholesterol levels in both strains. Plasma triglyceride levels were significantly elevated in the obese SS^LepR^mutant rats compared to the values measured in the lean SS rats (1193 ± 256 and 89 ± 16 mg/day, respectively) (Figure 1B). Chronic treatment with gemfibrozil markedly reduced the levels of plasma triglycerides in SS^LepR^mutant rats by more than 50% while not having any effect in SS rats (410 ± 79 and 32 ± 9 mg/day, respectively).

**FIGURE 2:**
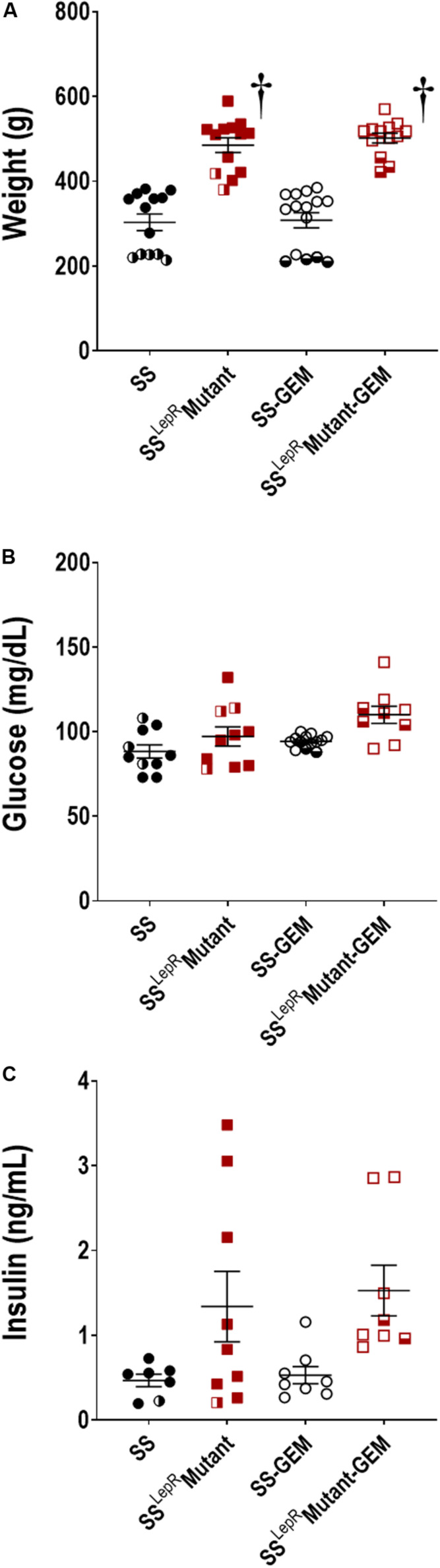
Comparison of metabolic parameters in wild-type Dahl salt-sensitive (SS) rats and the obese SS leptin receptor mutant (SS^LepR^mutant) strain treated with gemfibrozil: body weight **(A)**, blood glucose **(B)**, and plasma insulin **(C)**. Numbers of rats studied (*n* = 7–15 per group). Female rats in each group are represented by partially filled symbols. Values are means ± SE. ^†^*p* <0.05 vs. SS rats within the same treatment.

Comparison of body weight, blood glucose, and plasma insulin levels in SS and SS^LepR^mutant rats are presented in Figure 2. Body weight was markedly increased in the SS^LepR^mutant strain (485 ± 17 g) compared to the values measured in SS rats (303 ± 20 g) (Figure 2A). Chronic treatment with gemfibrozil had no effect on body weight in both strains. A hallmark characteristic of the SS^LepR^Mutant strain is the development of insulin resistance and obesity in the absence of hyperglycemia. In the current study, we did not observe any differences in blood glucose levels among the groups by the end of the study (Figure 2B). When examining plasma insulin levels at the end of the study, we found plasma insulin levels to be higher and more variable in the SS^LepR^mutant strain vs. the levels measured in SS rats (1.34 ± 0.42 and 0.47 ± 0.07 ng/mL, respectively), but the difference did not reach statistical significance (Figure 2C). This may be due to apoptosis of the insulin producing cells of the pancreas in the SS^LepR^mutant strain. Similar to body weight and blood glucose levels, treatment with gemfibrozil had no significant effect on plasma insulin levels in either strain.

### Cardiovascular and Renal Injury Parameters

**FIGURE 3:**
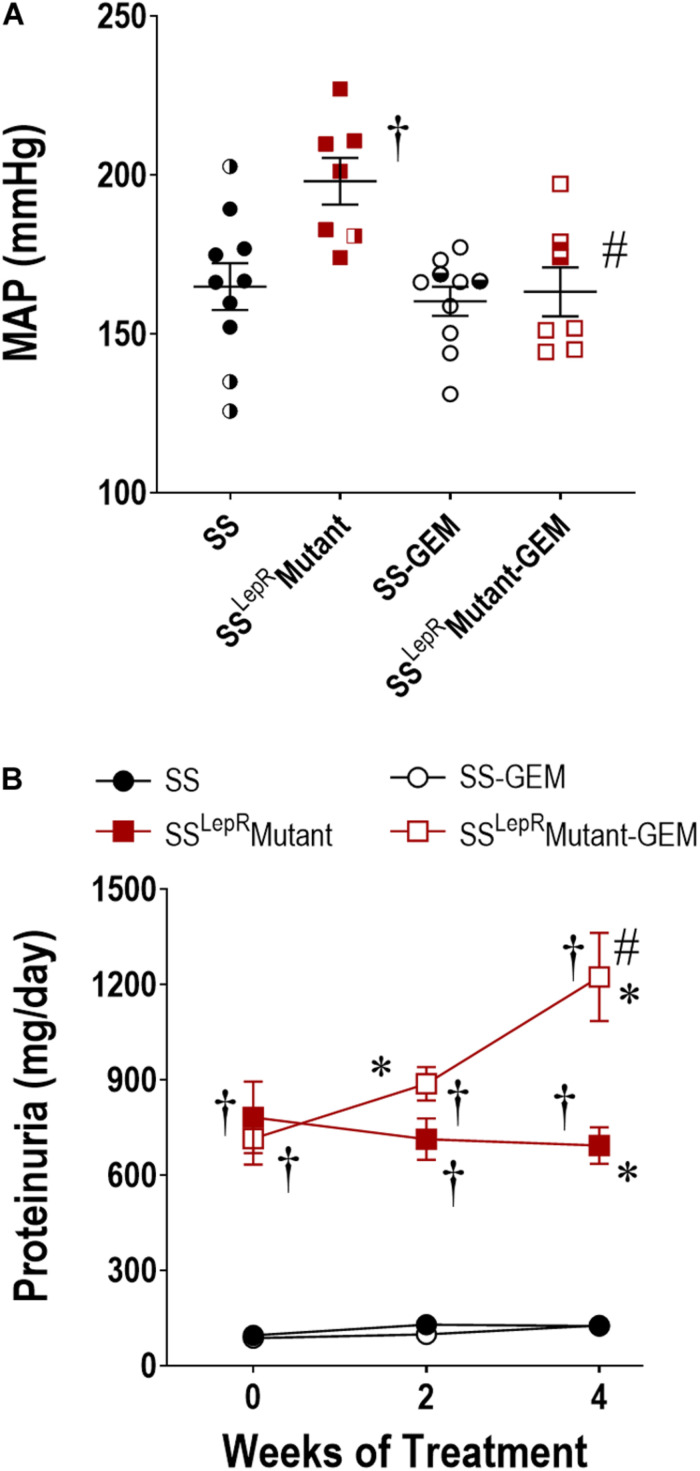
Effects of gemfibrozil treatment on mean arterial pressure (MAP) **(A)** and proteinuria **(B)** in wild-type Dahl salt-sensitive (SS) rats and the obese SS leptin receptor mutant (SS^LepR^mutant) strain. Numbers of rats studied (*n* = 7–12 per group). Female rats in each group are represented by partially filled symbols. Values are means ± SE. *p <0.05 vs. same strain at baseline, ^†^*p* <0.05 vs. SS rats within the same treatment, and ^#^*p* <0.05 vs. vehicle-treated rats within the same strain.

Comparison of MAP and temporal changes in proteinuria in SS and SS^LepR^mutant rats are presented in Figure 3. At the end of the study, MAP was markedly elevated in SS^LepR^mutant rats when compared to SS rats (198 ± 7 and 165 ± 7 mmHg, respectively) (Figure 3A). Administration of gemfibrozil significantly lowered MAP in SS^LepR^mutant rats (163 ± 8 mmHg) while not having any effect in SS rats (160 ± 5 mmHg). During the course of the study, proteinuria increased from 96 ± 13 to 125 ± 25 mg/day in SS rats but showed a trend to decrease from 783 ± 113 to 693 ± 58 mg/day in the SS^LepR^mutant strain (Figure 3B). Interestingly, treatment with gemfibrozil increased the progression of proteinuria by 77% in the SS^LepR^mutant strain (1224 ± 139 mg/day) without affecting proteinuria in SS rats (127 ± 13 mg/day).

**FIGURE 4:**
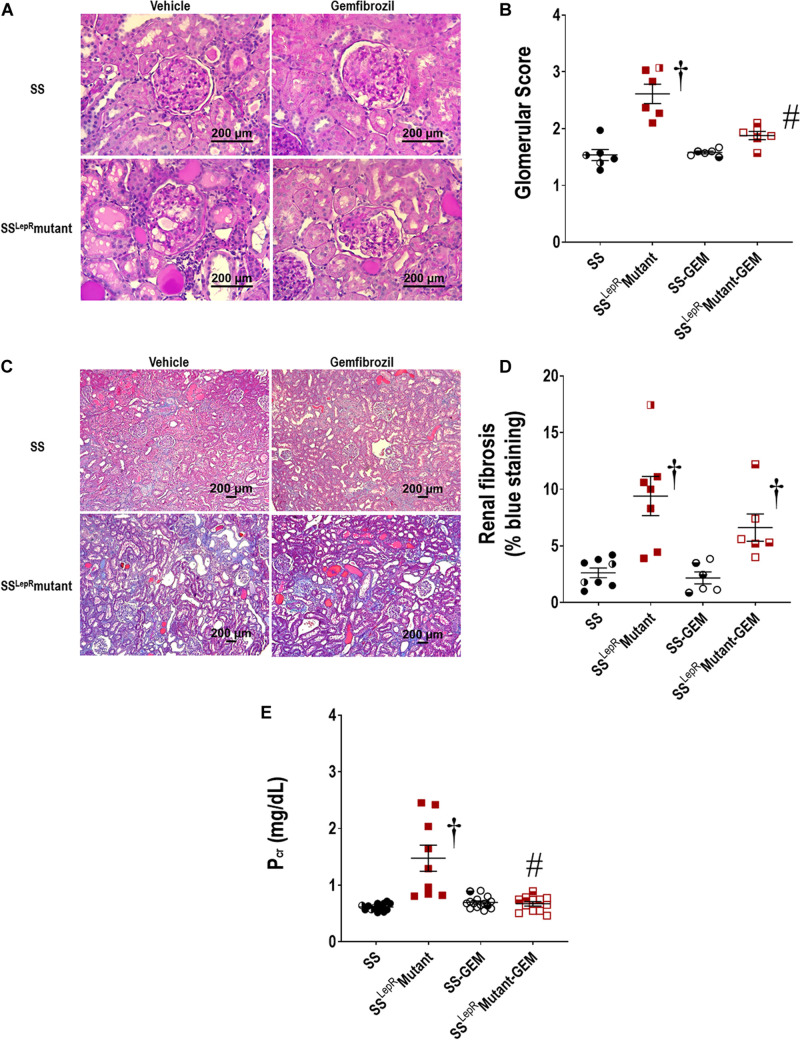
Effects of gemfibrozil treatment on renal histopathology and renal function in wild-type Dahl salt-sensitive (SS) rats and the obese SS leptin receptor mutant (SS^LepR^mutant) strain. Representative images of renal histopathology: Glomerular injury – Periodic acid-Schiff staining **(A)** and glomerular injury score **(B)**; Fibrosis – Masson’s Trichrome staining **(C)** and renal fibrosis (% blue staining) **(D)**. Measurement of renal function by plasma creatinine levels among the groups is represented in **(E)**. Numbers of rats studied (*n* = 6–12 per group). Female rats in each group are represented by partially filled symbols. Values are means ± SE. ^†^*p* <0.05 vs. SS rats within the same treatment, and ^#^*p* <0.05 vs. vehicle-treated rats within the same strain.

The effects of gemfibrozil on the degree of renal injury in SS and SS^LepR^mutant rats are presented in Figure 4. The kidneys from vehicle-treated SS^LepR^mutant rats exhibited more mesangial expansion and glomerular injury compared to SS rats (Figures 4A,B). In contrast, kidneys from the SS^LepR^mutant strain treated with gemfibrozil displayed reduced expansion of the mesangial matrix and glomerular injury. When examining renal fibrosis by thresholding for blue staining, fibrosis (% of blue staining) was significantly elevated in vehicle-treated SS^LepR^mutant rats when compared to the values measured in SS rats (Figures 4C,D). However, chronic treatment with gemfibrozil did not reduce renal fibrosis in the SS^LepR^mutant strain. When evaluating renal function, we observed that Pcr levels were considerably higher in SS^LepR^mutant rats vs. the values measured in SS rats (1.35 ± 0.21 vs. 0.62 ± 0.02 mg/dL, respectively) (Figure 4E). Moreover, administration of gemfibrozil improved renal function and markedly reduced Pcr levels in the SS^LepR^mutant strain without affecting the levels in SS rats (0.73 ± 0.07 vs. 0.70 ± 0.03 mg/dL, respectively).

### Renal Lipid Accumulation

**FIGURE 5:**
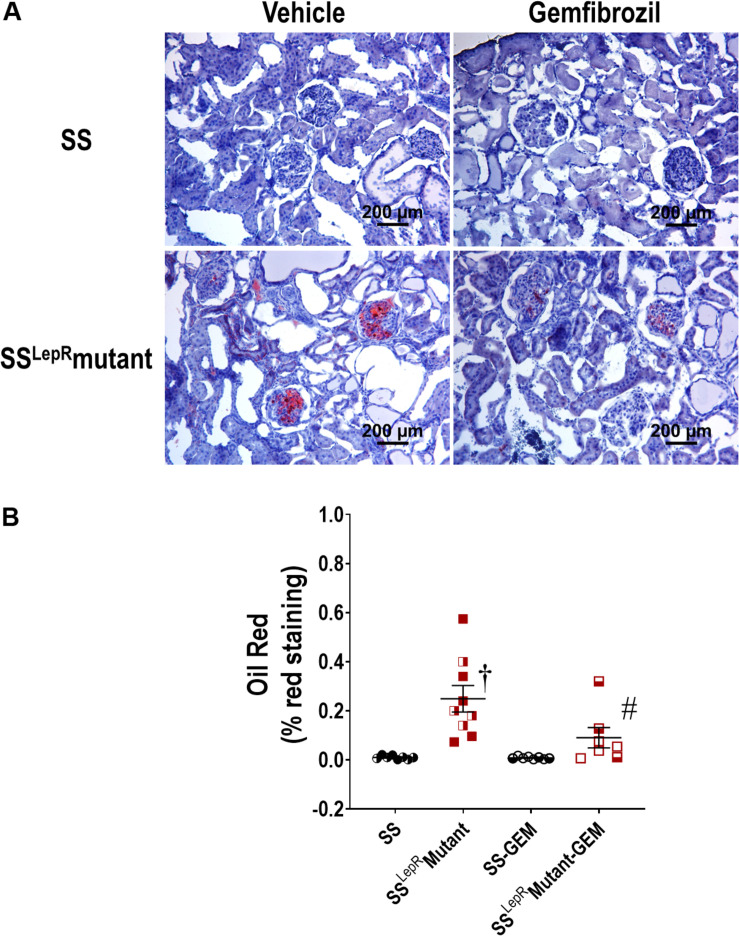
Effects of gemfibrozil on renal lipid accumulation. Representative images of renal lipid accumulation by oil-red-O staining **(A)** and quantification of % red staining **(B)** in the kidneys from wild-type Dahl salt-sensitive (SS) rats and the obese SS leptin receptor mutant (SS^LepR^mutant) strain. Female rats in each group are represented by partially filled symbols. Values are means ± SE. ^†^*p* <0.05 vs. SS rats within the same treatment, and ^#^*p* <0.05 vs. vehicle-treated rats within the same strain.

**FIGURE 6:**
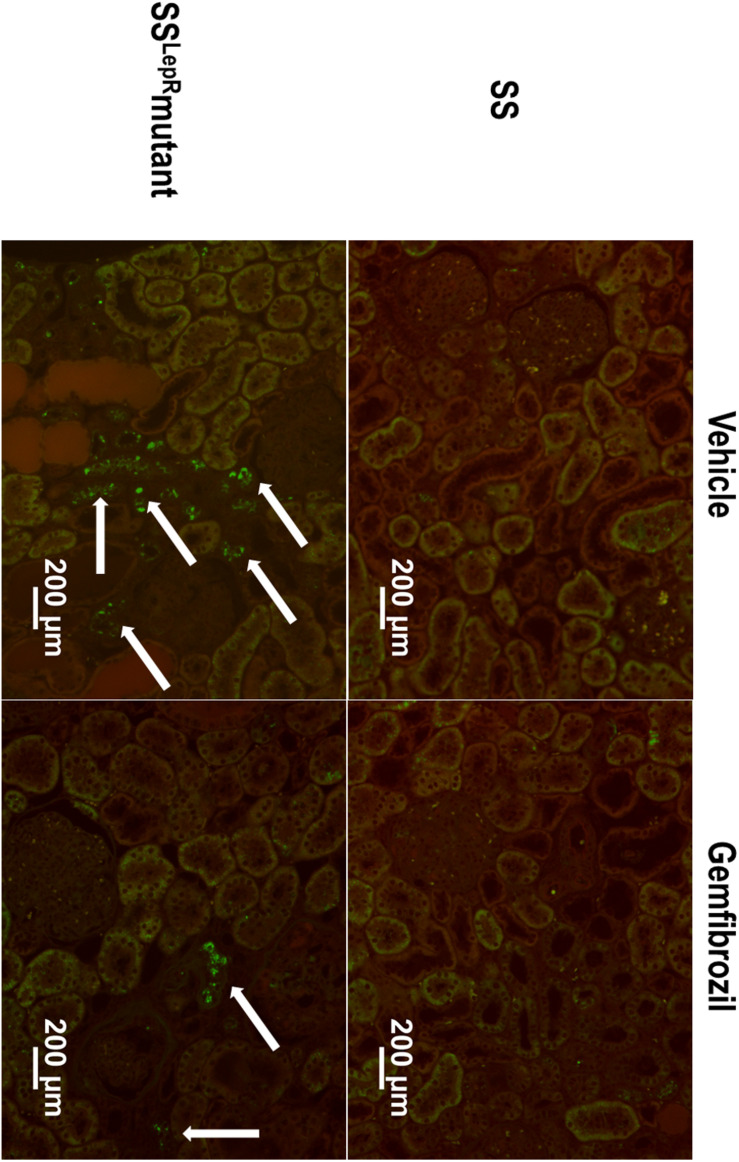
Effects of gemfibrozil on renal low-density lipoprotein (LDL) accumulation. Representative images of renal LDL accumulation by FITC-labeled BODIPY FL LDL staining (indicated by white arrows) in the kidneys from wild-type Dahl salt-sensitive (SS) rats and the obese SS leptin receptor mutant (SS^LepR^mutant) strain.

The effects of gemfibrozil on renal lipid accumulation via oil-red-O staining and FITC-labeled BODIPY FL LDL are presented in Figures 5, 6. We observed noticeably more oil-red-O staining in glomeruli, tubules, and interstitial space in vehicle-treated SS^LepR^mutant rats compared to SS rats suggesting significantly higher lipid accumulation in the kidneys of SS^LepR^mutant rats (Figures 5A,B). Chronic treatment with gemfibrozil decreased oil-red-O staining in SS^LepR^mutant rats. When assessing LDL accumulation by FITC-labeled BODIPY FL, we found similar results as with the oil-red-O staining, but LDL accumulation was more localized to the interstitial space in the vehicle-treated SS^LepR^mutant strain vs. SS rats (Figure 6). Moreover, treatment with gemfibrozil appeared to reduce LDL accumulation in SS^LepR^mutant rats.

## Discussion

One of the major characteristics of obesity is dyslipidemia, which has been linked to the development of CKD (Kasiske et al., 1993; Guijarro and Keane, 1996; Keane, 2000). However, the relationship between dyslipidemia and the progression of renal disease has been conflicting (Samuelsson et al., 1997; Hadjadj et al., 2004; Dalrymple and Kaysen, 2008; Rahman et al., 2008; Kaysen, 2009; Navaneethan et al., 2009; Chawla et al., 2010) and needs further investigation. Recently, we reported that the development of CKD in the SS^LepR^mutant strain was associated with dyslipidemia and renal lipid accumulation (McPherson et al., 2016, 2020). In the current study, we examined the effects of gemfibrozil, a lipid-lowering drug, on the progression of renal injury in SS^LepR^mutant rats during the progression of renal injury. Treatment with gemfibrozil had no effect on body weight, blood glucose, or plasma insulin levels in both strains. Plasma triglyceride levels were markedly elevated in the SS^LepR^mutant strain compared to SS rats, and chronic treatment with gemfibrozil reduced plasma triglycerides by more than 50% in the SS^LepR^mutant strain. Arterial pressure was significantly elevated in the SS^LepR^mutant strain vs. SS rats, and administration of gemfibrozil prevented the increase in arterial pressure observed in SS^LepR^mutant rats. We observed a trend for proteinuria to decrease in the SS^LepR^mutant strain over the course of the study, which was accompanied with significant glomerular injury and renal lipid accumulation (i.e., oil-red-O staining and LDL fluorescence) and fibrosis. Moreover, the concentration of Pcr was significantly elevated in the SS^LepR^mutant rats compared to the values measured in their lean SS control counterparts suggesting that the SS^LepR^mutant rats developed CKD. Chronic treatment with gemfibrozil markedly increased proteinuria, reduced glomerular injury, and improved renal function in SS^LepR^mutant rats. These data indicate that reducing plasma triglyceride levels with gemfibrozil reduces renal lipid accumulation and inhibits the progression of hypertension and CKD associated with obesity in SS^LepR^mutant rats.

Fibrates are agonists for the nuclear transcription receptor, PPAR-α, and are often used to effectively reduce plasma triglyceride levels by increasing the lipolysis of lipoprotein triglyceride via lipoprotein lipase (Heller and Harvengt, 1983; Keech et al., 2005; Remick et al., 2008). Additionally, fibrates are the most commonly prescribed drug for patients suffering from hypertriglyceridemia. In the current study, we chose to use gemfibrozil since it has less of an effect on stimulating a decline in renal function in patients susceptible to the development of CKD (Broeders et al., 2000). We observed that plasma triglyceride levels were markedly elevated in the SS^LepR^mutant strain vs. the values measured in SS rats, and treatment with gemfibrozil significantly decreased plasma triglyceride levels by more than half in the SS^LepR^mutant strain. Treatment with gemfibrozil was specific in lowering triglyceride levels because it did not have an effect on plasma cholesterol levels. These data suggest that the dose of gemfibrozil used in the current study was effective in reducing triglyceride levels in our rodent model of obesity with severe renal disease.

Obesity has been associated with elevated proteinuria in humans as well as animals (Bagby, 2004; Chen et al., 2004; Kurella et al., 2005; Kanauchi et al., 2006; Ninomiya and Kiyohara, 2007; Wahba and Mak, 2007; McPherson et al., 2016, 2019, 2020; Kovesdy et al., 2017). In the current study, proteinuria was already markedly elevated at baseline in SS^LepR^mutant rats compared to SS rats and had a tendency to decrease over the course of study. This result is not at all surprising, since we have previously observed that proteinuria declines after 14 weeks of age in the SS^LepR^mutant strain, which is more than likely due to a decline of renal function (McPherson et al., 2016). The impact of dylipidemia on the progression of proteinuria and decline in renal function is unclear but undoubtedly involves inflammation, oxidative stress, and lipid accumulation (D’Agati et al., 2016; Yang et al., 2017; Gai et al., 2019). While we did not measure inflammation and oxidative stress in the current study, lipid accumulation was significantly higher in glomeruli, tubules, and interstitium of obese SS^LepR^mutant rats compared to their lean counterparts. Various cells in the glomerulus (i.e., podocytes and mesangial cells) are sensitive to lipid accumulation, and once lipids affect these cells, they contribute to structural and functional changes that lead to the development glomerulosclerosis and proteinuria (Straub et al., 2013; de Vries et al., 2014), which eventually leads to decreased renal function and CKD. Interestingly, treatment with gemfibrozil for 4 weeks significantly increased proteinuria and reduced glomerular injury and renal fibrosis in SS^LepR^mutant rats without having any effect in SS rats, and this was associated with reduced renal lipid accumulation. We hypothesize that the increase in proteinuria is due to improved renal function observed in gemfibrozil-treated SS^LepR^mutant rats. In support of our hypothesis, Wilson et al. demonstrated that treatment with fenofibrate markedly increased proteinuria but reduced mesangial expansion and glomerulosclerosis in SS rats fed a high salt diet with pre-existing renal injury (Wilson et al., 1998). Taken together, these data provide direct evidence that reducing hypertriglyceridemia and renal lipid accumulation improves renal function during the progression of renal injury associated with obesity.

Previous studies have demonstrated that a major side effect of fibrates is decreased renal function (Broeders et al., 2000; Tonelli et al., 2004). This may be attributed to the potential inhibitory effects of fibrates on the production of vasodilatory prostaglandins in the kidney that regulate renal blood flow and glomerular filtration rate (Wilson et al., 1995; Ledwith et al., 1997). In the current study, we used gemfibrozil, which appears to be free from these renal dysfunction effects (Yoshinari et al., 1998; Broeders et al., 2000). We observed that chronic treatment with gemfibrozil did not impair but rather improved renal function in our model of obesity susceptible to CKD. Our results are very similar to what has been demonstrated in clinical studies (Broeders et al., 2000; Tonelli et al., 2004). Broeders et al. conducted a study that examined the effects of four main fibrates (i.e., fenofibrate, bezafibrate, ciprofibrate, and gemfibrozil) on renal function in patients with pre-existing renal dysfunction and found that all the fibrates except for gemfibrozil impaired renal function (Broeders et al., 2000; Tonelli et al., 2004). These results further suggest that gemfibrozil does not worsen renal function in patients or animals that are susceptible to CKD.

One of the major findings in the current study is chronic treatment with gemfibrozil significantly reduced arterial pressure in the obese SS^LepR^mutant strain during the progression of renal injury. The arterial pressure lowering effect of fibrates in various animals of models of hypertension and renal disease has been well-documented (Wilson et al., 1998; Cruz et al., 2011; Lee et al., 2011; Li et al., 2014; Weng et al., 2014). However, studies examining the effects of fibrates on arterial pressure in animal models with either established hypertension and/or renal disease are limited. Roman and colleagues reported that administration of fenofibrate decreased arterial pressure in SS rats fed high salt diet with established hypertension and renal disease (Wilson et al., 1998). The exact mechanism by which fibrates lower arterial pressure is not completely understood but involves various pathways such as anti-lipidemic (Jonkers et al., 2001; Kim and Kim, 2020), anti-inflammatory (Feingold and Grunfeld, 2000; Diep et al., 2004; Ruscica et al., 2018), nitric oxide (Newaz et al., 2005; Ibarra-Lara et al., 2010; Esenboga et al., 2019; Xu et al., 2019), and 20-HETE (Roman et al., 1993; Wilson et al., 1998). In the current study, we cannot exclude the impact of any of these pathways on reducing arterial pressure in response to gemfibrozil treatment. However, we hypothesis that the arterial pressure lowering effect of gemfibrozil is due to improving renal function in the obese SS^LepR^mutant strain. Moreover, to our knowledge, the current study is one of few studies demonstrating that treatment with a fibrate is beneficial in lowering arterial pressure and preventing the progression of CKD associated with obesity.

Overall, the results of the current study support the hypothesis that treatment with gemfibrozil reduces progression of renal injury in obese SS^LepR^mutant rats predisposed to the development of CKD. Yet, there are a few limitations that should be noted. One limitation is that we did not fully investigate the impact of sex in the current study. Previous studies have demonstrated that there are sex differences in the development of hypertension and renal injury in SS rats on either a high salt or high fat diet (Hinojosa-Laborde et al., 2000; Bayorh et al., 2001; Gerhold et al., 2007; Podesser et al., 2007; Turbeville et al., 2020; Zhang et al., 2020). In contrast to those previous studies, we did not feed our rats a typical high salt diet (4–8% NaCl diet) (Mohammed-Ali et al., 2017), but we will admit that the degree of proteinuria may be elevated in male vs. female SS rats on a lower salt diet containing 1% NaCl. However, other studies have shown no differences in the development of hypertension and renal injury between females and males in the SS strain on high salt diet (Hinojosa-Laborde et al., 2000; Murphy et al., 2012). In the current study, the susceptibility to hypertension and renal injury were similar in female and male SS^LepR^mutant rats, which suggests that inhibiting leptin signaling may temper sex differences in the SS rats. Another limitation is the measurement of arterial pressure by the chronic carotid catheter method, which was performed on the day after surgery and involves stress on the animals due being placed in constrainers for a given amount of time, instead of using telemetry. However, we were still able to observed differences in arterial pressure among the groups. If telemetry was used, temporal changes in arterial pressure could have been measured throughout the study rather than at the end. An additional limitation of the study is that we did not examine whether treatment with gemfibrozil had effects on lipid transporters in the kidneys. We recently reported that the progression of renal injury in the SS^LepR^mutant strain is associated with alterations in various lipid transporters that contribute to lipid accumulation (McPherson et al., 2020). Since the vehicle-treated SS^LepR^mutant rats developed progressive renal injury and CKD, we believed that the renal tissue from this group would be necrotic and not be reliable to use for the comparison of gene/protein expression of the lipid transporters to the other groups in the study. An additional limitation is that we did not test whether treatment with gemfibrozil would slow the progression of renal injury and improve renal function in SS^LepR^mutant rats with established impaired renal function, which may have produced different results. Future studies will be designed to consider these limitations.

### Clinical Translational Perspective

Over the last few decades, there has been a growing need to understand the development and progression of renal injury in obese patients in the absence of hyperglycemia (Wesson et al., 1985; Praga et al., 2000; Praga, 2002; Chen et al., 2006; Kramer et al., 2006). The obese SS^LepR^mutant strain represents an ideal model to study the role of dyslipidemia during the progression of CKD associated with obesity without the complications of hyperglycemia. Lipid-lowering drugs such as fibrates are commonly administered to patients suffering from dyslipidemia. However, examining the role of dyslipidemia as a mediator of CKD has yielded inconsistent results and still remains unclear. However, data from the current study have provided strong evidence that fibrates are advantageous for treating patients whom are suffering from dyslipidemia and susceptible to CKD. Over the course of the study, we observed that reducing plasma triglyceride levels with gemfibrozil leads to a decrease in renal lipid accumulation and prevention of renal injury and hypertension associated with obesity in SS^LepR^mutant rats. These results also support previous studies that demonstrate gemfibrozil does not further impair renal function in patients that are predisposed to the development of CKD (Broeders et al., 2000; Tonelli et al., 2004). This is contradictory to the other fibrates in this class of drugs, which have been shown to cause a reduction in renal function (Tannock, 2000; Kidney Disease Outcomes Quality Initiative Group, 2003; Harper and Jacobson, 2008). Overall, more studies are needed to test the roles of dyslipidemia and renal lipid accumulation in the progression of renal disease to be able develop novel therapeutic targets to prevent this devastating disease in obese patients.

## Data Availability Statement

The raw data supporting the conclusions of this article will be made available by the authors, without undue reservation, to any qualified researcher.

## Ethics Statement

The animal study was reviewed and approved by the University of Mississippi Medical Center Institutional Animal Care and Use Committee.

## Author Contributions

JW and CS provided conception and prepared the figures. CS, BP, KM, AB, UE, EB, LS, DC, and JW drafted, edited, and revised the manuscript, and approved the final version of the manuscript. All authors contributed to the article and approved the submitted version.

## Conflict of Interest

The authors declare that the research was conducted in the absence of any commercial or financial relationships that could be construed as a potential conflict of interest.
